# Identification of Thoroughbred Racehorse Welfare Issues by Industry Stakeholders

**DOI:** 10.3390/ani11051358

**Published:** 2021-05-11

**Authors:** Glen Mactaggart, Natalie Waran, Clive J. C. Phillips

**Affiliations:** 1 Centre for Animal Welfare and Ethics, School of Veterinary Science, University of Queensland, Gatton, QLD 4343, Australia; g.mactaggart@ihug.com.au; 2 Faculty of Education, Humanities and Health Science, Eastern Institute of Technology, Hawke’s Bay 4112, New Zealand; nwaran@eit.ac.nz; 3 Curtin University Sustainable Policy (CUSP) Institute, Kent St., Bentley, WA 6102, Australia

**Keywords:** animal welfare, thoroughbred racehorse, adaptive conjoint analysis, attitudes

## Abstract

**Simple Summary:**

The thoroughbred racehorse has unique welfare issues, being highly trained for racing, as well as regularly transported to race meetings. Following their racing career, retirement often requires racehorses to adopt a new lifestyle, with inherent risks to their welfare. Currently, there is little information on the most serious welfare issues confronting racehorses. We initially sought the opinion of a panel of experts, which provided 14 key issues that were then explored in more detail in a survey of stakeholders in the industry. We found that the key issues were the quality of horsemanship, horses’ health, educating horses to cope with the welfare challenges, and track and stable quality. Focusing on these issues will provide the best potential to improve welfare.

**Abstract:**

Identifying key welfare issues for thoroughbred racehorses could lead to an improvement in standards. A lack of scientific information on the relative importance of key issues was addressed by soliciting the views of, first, welfare experts in the industry and, second, a broader group of stakeholders, who selected the best welfare options by adaptive conjoint analysis. The experts represented racehorse breeders, veterinarians, trainers, owners, government officials, salespeople, farriers, transporters, and horse re-trainers for post-racing activities. In a focus group meeting, the experts identified fourteen key welfare issues, each with two to four levels that related to common husbandry practices. Then, in an internet survey, 224 stakeholders ranked the issues using adaptive conjoint analysis, in declining importance, as: horsemanship > health and disease > education of the horse > track design and surface > ventilation > stabling > weaning > transport > nutrition > wastage > heat and humidity > whips > environment > gear. Relatively uniform responses to the survey by the different stakeholder groups suggested that there was a common view on what had the biggest impact on welfare. An exception was a greater rating given by women than men to the importance of correct horse nutrition. The rating of importance for welfare of different levels of provision of each issue mostly conformed to the scientific evidence, with the exception of weaning. This understanding of the importance of welfare issues for thoroughbred racehorses could be used to target interventions to the most serious problems.

## 1. Introduction

The Thoroughbred racing industry is frequently challenged by the public on welfare and ethics issues, especially the high wastage rates of racehorses. In addressing the welfare issues, it is important to assess the ability of the TBR to adapt to a closely managed environment and the extreme work-loads, with the latter restricted by its flexibility in both behavioural and physical phenotype [1]. Furthermore, the social nature of the horse and its natural existence in a home range are also important, both of which are precluded when TBRs are kept isolated in stables. Stable design frequently prevents physical and visual contact between equines, and frequent turnover of TBRs make the group unstable [2]. Nutrition is very different from a natural feeding pattern, which produces regular food intake to support digestion in the gastrointestinal tract [3]. In stables, cereals usually are the main feed, offered in a small number of meals with a limited amount of hay [4].

Blood samples can be taken to provide information on the welfare of TBR but they are expensive, mainly because veterinarians are required to take samples because of the invasive nature. Traditionally cortisol measurements have been most used to provide some information on chronic stress in TBR but taking samples may in itself induce stress [5]. Numerous variables affect cortisol levels, such as age, environment, weather conditions, and the time of day. Novel indicators include measurements of the expression of genes related to inflammatory and immune responses [6,7,8]. There is a growing interest in finding less invasive methods by which to assess the welfare of horses. For example, by questionnaires of owners of non-racing horses and veterinary examinations [9], or by observation of health and behaviour parameters [10]. Particular promise has been shown with infrared thermography, especially of the eye [11], and the use of a pressure head collar [12]. With TBRs, different techniques are required to include their welfare on the racetrack compared with Arabs, which is affected by their locomotory, cardiovascular, and respiratory soundness [13].

In the absence of means of relating surveys to known welfare criteria, the Delphi method can be used, which collects and distils data from anonymous experts. This can use a series of data collection rounds and analysis methods, as well as providing feedback, in an iterative process. Unlike Classical Delphi, which ranks issues and seeks consensus from homogeneous groups of experts, Policy Delphi uses an informed group to consider all possibilities, without seeking a decision [14]. First it identifies a panel of experts [15], taking into account levels of expertise [16,17] and their occupation [18,19]. Experts must have extensive experience in the field, appropriate formal education or professional achievements in the industry and must represent all aspects of the industry without too much overlap [15].

Vignettes have been used successfully to describe equine welfare [20], and are recommended as they secure respondent engagement [21] and may avoid the ethical issues linked to sensitive issues [22] and reporting bias, in which respondents give answers sought by the researchers [23]. However, they are criticised for slow response times [24], but do allow respondents sufficient time for detailed consideration [25]. Welfare assessment systems must also take into account scientific evidence, not just the opinions of experts [15,17,18].

Currently, there is no valid reliable and feasible tool available to assess TBR welfare. Therefore, we used experts to help develop an online adaptive conjoint analysis survey, which investigated the opinions of TBR stakeholders on appropriate welfare indicators. Then we compared their responses with scientific literature on each welfare parameter.

## 2. Materials and Methods

### 2.1. Focus Group Meeting of Experts

An expert meeting was organised to determine the key welfare issues for TBR that could then be placed in priority order using a wider stakeholder survey. Invitations were sent to 15 national associations connected with TB racing industry in Australia, selected from the Australian Jockey Club web page, Aushorse, and Racing Journals from all Australian States. The nine accepting associations were requested to send up to two representatives to a meeting held at the University of Queensland, Gatton Campus, which was facilitated by one of us (NW). An outline of the overall project (which aimed to develop a welfare index for TB racehorses in Australia) was provided, together with an invitation to speak for five minutes in their relevant area. The following experts attended the meeting: Breeders (*n* = 2), Owners (*n* = 2), Veterinarians (*n* = 3), Sales personnel (*n* = 1), Farrier (*n* = 1), Transporter (*n* = 1), Trainer (*n* = 1), State Government official (Queensland Racing) (*n* = 1), Retraining/Show horse (*n* = 1).

The facilitator initially described welfare indices and their application in animal industries, then the industry experts considered the following key husbandry issues that emerged from their presentations: breeding, weaning, transport, racing, training, horsemanship, horse use post-racing, nutrition, stabling, health, disease, sales, environment, heat, humidity, ventilation, whips, and racing gear. For each key issue, up to four levels of husbandry provision were identified. The first of the levels represented the ideal husbandry situation for TBR welfare and the last represented the least desirable husbandry option.

### 2.2. The On-Line Stakeholder Survey

The issues and levels were used in the development of a stakeholder survey, which was distributed widely to those in the industry in Australia and aimed to quantify opinions on the most appropriate levels of each issue. The survey was constructed in Sawtooth Software^®^ (Sawtooth Software, Inc., Sequim, WA, USA), using the Adaptive Conjoint Analysis (ACA) module. Conjoint analysis is a statistical technique used for analysing stakeholder preference, which attempts to assess the impact of specific features on overall preference and determines which combination of a limited number of Levels of Issues presented concurrently is most preferred. The technique allows several factors to be considered jointly, as well as in isolation, assuming that complex decisions are not based on a single factor [26]. Through an algorithm, ACA determines the combination of issues most influential in determining each respondent’s selection.

Three to four levels were assigned for each issue, with positive and negative Level Importance Values (LIV) representing levels rated high or low by each respondent, respectively. A pilot study was undertaken with 10 stakeholders, who were individually interviewed. In response to their comments, minor changes were made and a second pilot survey undertaken, resulting in no further changes.

The survey consisted of four sections of questions [27], with the last three forming the ACA system; prefaced by a consent form and a description of the project. The first (demographic) section asked respondents to provide their gender, age category, highest level of education, length of practical experience with TBR, country in which experience was gained, and the length of their involvement with TBR. Respondents self-reported the capacity in which they were involved with TBR: breeder, farrier, owner, practising veterinarian, retrainers, transporter, trainer, racing administration, or sales personnel. Re-trainers undertake the rehabilitation of racehorses after racing into equestrian sports, polo, trail riding, showing, or as companion animals. In the second section, respondents ranked the issues in order of importance for TBR welfare, from “1” (most important) to “14” (least important). In the third section respondents rated each level of the issues in terms of its importance on a five-point scale. In the fourth section respondents rated a series of paired-comparisons using conjoint analysis. Each question comprised two vignettes, each with two different levels of the same two issues. The respondent was asked for the strength of their preference for one or other vignette, with the question being “if two Thoroughbred racehorse establishments are identical in all other ways, which is better for the Thoroughbred racehorses’ welfare?” Respondents answered using a 9-point scale ranging from strongly prefer left (vignette 1) to strongly prefer right (vignette 2). The levels of some issues were considered to be incompatible with other levels of different issues, and consequently six combinations were prohibited to avoid conflicts of opinion in the discriminant process.

The online survey of questions for each of the fourteen issues and levels within the issues was sent to 1773 stakeholders in Australia. Respondents were a convenience sample selected on the basis of their prior knowledge and awareness of the subject, sourced from Racing journals and web sites, Thoroughbred sale catalogues, Racing magazines, Thoroughbred Stud Farm breed catalogues, and telephone books. A link to the survey was sent by e-mail to stakeholders in the following 9 groups: 315 breeders, 793 trainers, 135 veterinarians, 37 transporters, 119 farriers, 93 sales, 245 racing administrators, and 10 TBR re-trainers. The e-mail addresses of 26 owners were not available using these resources and were obtained by approaching them on race days and asking if they would accept to participate in the on-line survey and provide an e-mail address.

### 2.3. Statistical Analysis

Only completed surveys were included in the analysis, and a total of 179 incomplete surveys were discarded. The SMRT Market Simulator estimated Issue and Level Importance Values, using the parameters included in the conjoint analysis study. Level Importance Values were determined for all Levels of each Issue using the Simulator, which imported respondents’ level preferences into a hierarchical Bayes model using a Monte Carlo Markov chain algorithm. This used data from the population (means and covariances) to describe the preferences of individuals who had made a limited number of choices. The Level Importance Value data were normalised across respondents by zero-centering the utility values within each issue, i.e., the sum was equal to zero. The data were used to estimate the probability distribution of the parameters using conditional probability techniques at different levels. Ratios were then used for determining the Issue Importance Values; thus 10% is twice as important as a value of 5%.

General linear models were used to test for the effect of demographics (age, gender, level of education, stakeholder group, experience, and country where experience was gained) on the Issue Importance Values, but the residuals were not normally distributed. Attempts to manipulate the data mathematically still did not produce normally distributed residuals, and Moods median nonparametric statistics were used to the significance of differences between median [28]. The demographic results for the question relating to the respondents’ length of practical experience indicated a need to collapse the first two categories of 1–6 months (2 respondents) and 7–12 months (2 respondents) into one category, as there were less than 6 respondents in the two categories. Consequently, the reanalysis included three categories for experience: 1–12, 13–48 and >48 months.

## 3. Results

### 3.1. Focus Group Meeting of Experts

The fourteen key husbandry issues identified at the expert meeting and selected for the online survey were: horsemanship, weaning, stabling, environment, heat and humidity, ventilation, transport, wastage, gear, track design and surface, health and disease, education of the horse, whips, and nutrition (Table 1).

### 3.2. The On-Line Stakeholder Survey

A total of 403 surveys were returned, of which 224 were complete, representing an overall response rate of 23% and completed survey response rate of 13%. The highest response rate was from trainers (25%) and the lowest response rate from TBR transporters (2%) (Table 2).

There was a small majority of respondents that were male (57%), and most respondents were aged between 41 and 60 years (Table 3). When asked to indicate the highest level of education achieved, about half indicated they had a University degree, and the other half was evenly split between school leavers and graduates from Technical and Further Education colleges. The most common primary occupations in the TBR industry were trainers, veterinarians and breeders, with transporters, farriers and re-trainers relatively uncommon. Over 90% had more than four years of experience with TBR.

The median Issue Importance Value for each of the 14 issues ranged from 2.8 to 8.8 (Table 4). The issues ranked in order of the most to least important, with differing superscripts indicating a significant (*p* < 0.05) difference between issues, were: horsemanship ^a^ > health and disease ^ab^ = education of horse ^ab^ > ventilation ^b^ > track design and surface ^c^ = stabling ^c^ = weaning ^c^ = transport ^c^ = nutrition ^c^ > wastage ^cd^ = heat and humidity ^cd^ > whips ^de^ = environment ^de^ > gear ^f^.

There were no significant effects of stakeholder group on Issue Importance levels, except for the issues Weaning and Nutrition, which tended to be rated less important by the re-trainers (6.7 and 6.4, respectively), farriers (7.2 and 5.9, respectively), and veterinarians (Nutrition only, 6.5), than the other stakeholder groups, whose Issue Importance Levels ranged from 7.0 to 8.0 for Weaning and 7.1–9.1 for Nutrition (for all of their Importance values see Appendix A Table A1) (Weaning *p* = 0.02 and Nutrition *p* = 0.01). Gender only significantly influenced the Issue Importance Values for Nutrition, which was rated more important (7.9) by females than males (7.1) (*p* = 0.007), whereas there was a tendency for males to rate track design of more importance (7.8) than females (7.2) (*p* = 0.08). Respondent age only influenced the rating of Health and Disease, the importance of which declined with age (years), 25–30: 9.7, 31–40: 9.1, 41–50: 8.4, 51–60: 8.2 and 61+: 8.1 (*p* = 0.03). Respondents with little experience of TBR tended to rate the horses’ Environment and Gear as more important, whereas those with longer experience tended to rate Horsemanship, Education of the horse, Transport and Nutrition as more important (Table 5). Level importance values are reproduced in Appendix A Table A2, with the exception of Weaning.

## 4. Discussion

This study was the first undertaken to establish a uniform ranking system of the issues important for the welfare of TBR, using expert opinion and a stakeholder survey. This combination of methods and wide variety of stakeholders was thought necessary because TBR are subjected to a variety of management and housing practices throughout Australia. The high response rate by owners was probably due to them being individually approached by the researcher for their email addresses, which are not usually available on the internet, nor do owners have a web page, and owners’ addresses are not available from Race Clubs of TBRs, Racing Journals, Thoroughbred sales catalogues, or Thoroughbred Stud Farm breed catalogues. The next highest response came from the TBR Re-trainers, which may indicate the perceived importance of reducing wastage of TBRs.

Social structure normally defines relationships between individuals through competition, cooperation, dominance, social units, care of offspring, and use of resources [29], but this important issue was not raised at the Expert meeting perhaps because the experts felt it to be already included in other issues, such as Environment. We assume experts were aware of its importance and we therefore discuss this and the issues actually raised in the context of information in the scientific literature.

### 4.1. Social Structure

The horse is a prey animal whose natural choice of social living is a means of self-preservation, together with speed and agility [30]. An appropriate social structure exists in all mammalian species in order to meet social, reproductive and psychological needs [31,32]. The feral or free ranging horse is demonstrably social, forming “bands” or social units typically consisting of 1–3 mares, their offspring, and an adult harem stallion [32]. They may grow to 20 animals [33]. Group size can be a response to the environment as well as population density [32]. Lone or small bachelor groups account for the remaining males in a population, and occasionally solitary aged females may be sighted. The basic composition and organization of a primary group rarely changes [34], with adult males usually at the top of feral groups [35] and mares at the top if humans restrict the number of stallions [36].

### 4.2. Horsemanship

The importance of qualified staff was given the highest ranking by the stakeholders, particularly by those with more experience of TBR’s. The human horse relationship is based on good knowledge of husbandry, skilled handling, care treatment, and an affinity and empathy with animals, dedication and patience. The most important times for this relationship to influence welfare is during training and competition. In Arabian horses, stress responses after race training are less than after competition, but the starting gate does not impose any additional stress [37].

### 4.3. Health and Disease

The Stakeholders ranked this extremely important issue second, probably because training and management methods frequently cause physiological problems, which contribute to a deterioration in health as well as the immune system. For example, horses that are transported either by road or air are prone to respiratory tract infections with viruses, bacteria and mycoplasmas [38]. During training the horse adapts through long-term adjustments to its oxygen transport capabilities, as well as inflammatory cytokine gene expression upregulation [39] the tasks required of Thoroughbred racehorses must be within their natural range of capabilities.

Housing, exercise, and feeding of TBR differ from the social structure, behavioural ecology, and activity budgets of feral and free ranging horses, producing performance-related clinical problems as a result of intensive management, stable design, nutrition, training methods, and transportation. The result may be compulsive obsessive behaviour. At its worst, stress-induced abnormal behaviour is dangerous to all concerned, especially between human and animal when the horse is being ridden [40,41].

Recovery time after a period of race training [42] determines whether sufficient bone remodelling occurs—chronic fatigue can develop if the horse is over raced [43]. Over racing causes adverse behavioural and physical responses which resting for several weeks does not correct. This syndrome [44] is best detected by regular monitoring of body weight and mood changes [43]. Bone remodelling occurs during the first 10 weeks of a rest period, which correlates with the usual length of time TBRs are in training during preparation [42].

### 4.4. Education of the Thoroughbred Racehorse

This ranked almost as high as Health and Disease, especially by those with long experience. Foundation training and learning theory applied consistently improves TBR safety and welfare and provides a solid base for continued training in other equestrian pursuits [43]. Early handling and training of the Thoroughbred foal through to adulthood will help to avoid fearful situations developing into problem behaviours. Foundation Training (previously “breaking-in”, which has negative connotations [43]) facilitates future training. Early handling by competent staff has welfare benefits [44], as exposing them to stimuli while they are relatively impressionable is effective [45], irreversible and remains for life [46]. Foals learn to tolerate rather than struggle, reducing defensive aggression [44,46]. Naturally complex environments develop foals’ learning ability and behavioural plasticity, allowing humans the scope to shape behaviours [44]. In Australia most foals develop in a low stress environment, in much the same way as a feral foal develops: i.e., pair bonds are formed, play is experienced, and allo-grooming is perfected. Experiencing a natural environment as foals may subsequently help the TBR to cope with stressful situations [47], such as being covered by a stallion on another breeding farm, long journeys or treatment at a veterinary clinic. Such stresses can develop into obsessive compulsive behaviour [45].

### 4.5. Track Design and Surface

The ranking of Track Design and Surface as being equally important as Stabling, Weaning, Transport, and Nutrition (Table 4) probably stems from lameness associated with poor track design and lack of surface care [48]. Insufficient track preparation can lead to sub-optimal performance, increased risk of injury, exhaustion, and even death [48]. Most injuries are track related [49], and shin sores are a major cause of lameness in young Thoroughbred racehorses in Australia, leading to retirement of animals from the industry [48].

Fractures are another common cause of wastage in Australia [48,49]. Most racehorses are trained on public racecourses where records of training injuries are not maintained, and are less visible to the public than those sustained on race day [50], which are recorded by veterinarians employed by the race club. More comprehensive recording of training injuries will facilitate improvement of racetrack design and maintenance [43]. Synthetic tracks are increasingly common, especially in drought areas where water shortages hamper the maintenance of the track. Very little is known about injuries sustained on synthetic tracks but the equitrack surface at least may be protective [50].

### 4.6. Ventilation

Stables compromise both physical and visual contact and frequently have poor circulation and air quality, with high ammonia concentrations, and dust/moulds from hay, straw and bedding. The respiratory system of the horse is compromised [51] and obstructive pulmonary disease may ensue [52]. Huffman [51] suggested that adequate ventilation should be mandatory in all new stabling, including ceiling fans. High levels of dust endotoxins can lead to respiratory disease, whilst excessive air flow stimulates the sensitive nerves in the nose and can also be responsible for stereotypic behaviour such as head shaking.

### 4.7. Stabling

The normal behaviour of the horse is dramatically altered when stabled; social grouping is absent, exercise is limited, foraging does not occur, feed is concentrated (requiring a short time to consume), and time budgets altered so that boredom is a frequent outcome. Stereotypic behaviours are commonplace, believed by many to be coping mechanisms to environmental stressors. However, abrupt weaning practices are also a major cause of oral stereotypic behaviour, while locomotor stereotypic behaviour occurs most often when a horse is moved from the breeding farm to training stables [53]. Isolation, confinement, altered time budget, and concentrated feed all play a role in the development of stereotypic behaviour.

Long lines of loose boxes are the most common type of stabling in Australia, but they are being superseded by large barns where horses are housed in loose boxes under one large roof [54]. TBR housed in loose boxes may be completely enclosed on three sides, with the stable door the only opening.

The restricted environment of stables severely reduces the normal pattern of locomotion. Free-ranging or feral horses take many thousands of paces per day when grazing. Thus stabling of Thoroughbred racehorses and feeding of a high grain ration can cause many welfare problems, particularly the enforced immobility (sometimes stable confinement is as much as 90% of the day) followed by intense physical activity, frequently resulting in the painful muscle syndrome Azoturia (exertional rhabdomyolysis) [55]. The resultant isolation and boredom triggers stereotypic behaviour, such as box walking and/or weaving, crib biting, wind sucking, wood chewing, and licking [56]. Various devices are employed to prevent, but never cure, abnormal behaviour [57].

### 4.8. Weaning

Weaning is extremely stressful for both mother and foal, with many neonate oral stereotypies commencing at this time; gradual weaning of foals being less stressful than abrupt weaning [58]. The re-trainers and farriers rated Weaning as a less important issue for the welfare of TBR than other stakeholders. Neither are dealing with weaning, so this is not surprising.

In our survey stakeholders preferred “two weanlings isolated together in a stable which allows visual and physical contact with neighbouring horses”. This method is time consuming because the weanlings then have to be weaned from each other; there is a tendency for bullying, and sometimes inter sucking occurs [59]. However, findings of a study [58] of foals weaned between 3 and 9 months found less incidence of oral stereotypic behaviour when “one mare is removed at a time from a group of mares and foals (in a paddock) over a period of weeks until all mares are removed from the group”. Other studies [60,61] support these findings.

Diet at weaning may also have far reaching effects: grain supplements fed prior to weaning contribute to the development of stereotypic behaviours [58], supporting a relationship between diet and oral stereotypy in adult stabled horses [4,61,62,63]. Use of a pasture supplement contributing fat and fibre is advocated [4], to reduce stereotypic behaviour and gastrointestinal ulcers.

The weanling is subjected to more intensive handling as it develops and is prepared for sale as a yearling. If harsh methods are used and/or the yearling becomes confused the handling becomes a welfare trigger. When learning to lead, the yearling experiences the first lessons in classical conditioning and negative reinforcement, the beginning of foundation training for all further training [43].

### 4.9. Transport

Transportation of horses increases their heart rate and energy expenditure [64] and contributes to illnesses such as acute colitis, laminitis, transit tetany, trailer choke, and mild azoturia [4]. Horses have long been transported over long distances by sea transport (once the only method available, [65]). The associated Shipping Fever is a combination of pleurisy and pneumonia. In the 19th century rail travel became increasingly popular for moving horses but it is now common to fly racehorses to interstate race meetings. Dehydration and weight loss are risks in all long distance transport, as is Shipping Fever caused by a horse’s head being tied in such a way as to prevent it being lowered or raised, thus preventing effective draining of the upper respiratory tract [52]. Shipping fever is also a major concern with air transportation [66].

There are many areas where the risk of injury to horses during transport can be reduced. Horses facing the rear of the vehicle have minimal loss of balance, carry their heads lower, make less contact with the sides of the vehicle and show a less rigid body position than when forward facing [67]. Familiarising horses with one another before transport, with frequent rest and water breaks during the journey is recommended [68]. Horses transported either by road or air are prone to respiratory tract infections, including viruses, bacteria, and mycoplasmas.

Thoroughbred racehorses are transported many times in their life, and many do not show transport stress. However, some suffer a negative experience such as a fall, unfamiliar orientation, or short head ties, which leaves the animal fearful and may result in poor loading behaviour.

Performance may be compromised when journeys are long i.e., 24 h or more [69]. Some horses are less stressed by transportation than others. Other influences which can impact on welfare during transportation are the care, skill and experience of the driver, ventilation of the vehicle and road surface. Today many training establishments are located on racecourses, therefore reducing the need for road transport, which may be why transport only received a middle to low rating in our survey.

### 4.10. Nutrition

The horse, as a non-ruminant herbivore, is suited to a high fibre, low starch diet, but this is unable to fulfil the energy requirements of the TBR [70]. Twice as much energy should be supplied from roughage than from concentrates [71]. The digestive system of the TBR can also cause welfare problems in the intensively managed stabled TBR with decreased gastrointestinal function, which often results gastric ulcers, colic and laminitis [72]. Feral or free ranging horses spend between 50% and 70% of their day grazing [32] while TBRs are fed mainly high energy concentrated grain diets, which may take only 15% of their time to consume [30]. Reduced gastrointestinal function leads to painful conditions such as colic, laminitis, and gastric ulcers [72], inducing stereotypic behaviour in stabled TBRs.

Welfare problems stemming from poor nutrition are not universal, which explains its middle ranking in our survey. It is of interest that the re-trainers, farriers, and veterinarians rated Nutrition as a less important issue for the welfare or TBR. None of these are dealing with the feeding of TBR on a daily basis, nor would they necessarily be asked for advice on nutrition, this being the domain of nutrition specialists. Women rated nutrition as more important for the TBR than did men, perhaps reflecting the fact that women are often more concerned about dietary quality than men [73].

### 4.11. Wastage

Wastage is defined as “any injury or disease that involves an interference with the training schedule of a TBR resulting in lost days in work, and prolonged rest or retirement from racing.” Many TBRs are retired from racing at a young age, some TBRs never race, for reasons usually due to unsoundness, injury, unsuitable temperament, or insufficient speed. Most injuries are track related [47]. Efforts to reduce wastage due to injury and unsoundness by improving track design and surface can be complemented by improved training methods. In the Australian racing industry, orthopaedic problems are the main reason for wastage in TBRs [74]. An improvement in riding standards is also an important factor in lowering wastage rates aided by better training methods, and with the correct use of foundation training.

Wastage is also a welfare issue if a TBR is retired to a riding establishment or as a companion and then has poor welfare. Avoiding this may require improvements in foundation training [43], as well as using skilled horse-people to re-educate the TBRs prior to taking on their new role. Apart from this wastage can be seen as mainly an ethical issue, not a welfare issue, which explains its relatively low rating in our survey.

### 4.12. Heat and Humidity

Horses have a wide thermal tolerance (−10 to 30 °C [75], particularly tolerating cold well. Hence, it did not receive a high rating in our survey. Acclimatisation is important when managing equines in adverse weather conditions. Horses in Australia are more likely to experience hot than cold stress and are often seen using shaded areas, natural wind breaks, sun baking and even wading in water to regulate body temperature.

### 4.13. Whips

In Thoroughbred racing both frequency of usage and design of whips is controlled in most jurisdictions. Even so, the psychological pressure and pain inflicted by whips may have a detrimental effect on the horse, shortening the stride [76]. A significant proportion of breakdown injuries occur after using the whip [77], in part because the horse’s rhythm is destroyed. Padded whips may not have their intended consequence of reducing pain, and overuse of any whip can produce stress induced analgesia [78]. There is, therefore, doubt about the functionality of whips in races, which may explain why it got a relatively low rating in our survey.

### 4.14. Environment

The stable environment for the TBR is important in removing pollutants and contributing to comfort. Most Australian Racing bodies regularly inspect stables to see if they are compliant with Animal Welfare Policies of their respective States. This may explain why the environment received a low rating by our respondents.

Training requirements frequently demand that TBRs are exercised in the early morning dawn, with subsequent confinement indoors for nearly all of the day. Lack of vitamin D is compounded by stress on the vertebral column from the weight of the rider and intensive training increasing the amount of bone remodelling [79].

Yards can be as isolating for equines as stables, but when well designed can allow visual, physical and auditory contact with adjoining equines, as well as a choice of environments. Of the many bedding types used, straw, wood shavings, shredded paper, cardboard, and sand, TBRs show a preference for straw bedding [80].

### 4.15. Gear

Gear was consistently rated as the least important issue in our survey, and those with less experience rated it higher. It includes saddles, stirrups, bits, and other tackle, and it is to be expected that the inexperienced will focus on such practical issues, rather than the more amorphous issues of horsemanship and education of the horse. Back pain is a common cause of poor performance and is usually the result of poorly fitting saddles [81], while girth tensioning can cause pain and discomfort and decrease inspiratory air flow or volume [82]. The horses’ mouth is extremely sensitive and for this reason should be treated with great care when making the connection between horse and rider. The response of the horse to the rider’s signals is an important part of a young horse’s education, thus it is important for the rider to give clear signals [83]. Fortunately, most racehorses are ridden with the simplest and kindest of bits [83], a snaffle. Tongue ties, tongue clips and some nose bands are allowed in order to prevent the horse getting its tongue over the bit or swallowing its tongue during racing approved by the Australian Racing Board [84]. One type of bit prevents horses from getting their tongues over the bit or hanging outside the mouth and also stops horses from pulling and bolting, potentially eradicating head shaking and preventing displacement of the soft palate [85]. Blinkers are used to preventing shying or bolting, to increase the animals’ focus and avoid their fear of horses racing close to them. It is this functionality of gear in protecting the horse and rider that explains why it received the lowest rating by our respondents.

### 4.16. Limitations of the Study

The welfare issues posited were necessarily general and rather superficial, because otherwise they might not have been relevant in some circumstances. The level of specificity might have affected the ranking of levels. Some, e.g., health and education of the horse, could only use a comparison between regular, some and infrequent attention to the issues, and individual respondent’s interpretation of that could vary considerably. Similarly, respondents’ interpretation of gradual and tight turns in the track is open to interpretation. By contrast, we were able to be more specific in relation to horsemanship, with all, 50% and no staff experienced and trained. The issues relating to specificity are well illustrated by our designation of stable sizes, which must relate to the size of horse being housed, in relation to welfare impact. However, such complexity could not easily be built into the questionnaire.

## 5. Conclusions

An initial survey of experts produced 14 key concerns about the welfare of TBRs, which were then explored in a Stakeholder survey. For the most part stakeholders in the TBR industry were consistent in their attitudes to the most important of the issues, rating horsemanship > health and disease > education of the horse > track design and surface > ventilation > stabling > weaning > transport > nutrition > wastage > heat and humidity > whips > environment > gear. However, ratings given to nutrition were lower in men than women and stakeholders with less experience tended to rate the use of gear and the horses’ environment as more important than horsemanship and education of the horse. The rating of which levels of each issue were better for welfare were mostly supported by a scientific assessment, with the exception of weaning. Stakeholders preferred isolation of pairs of weanlings in a stable, whereas the scientific literature suggests that gradual removal of mares from the paddock is best. The issues identified could, with further research, form the basis of a welfare index for the TBR.

## Figures and Tables

**Table 1:** Issues, Levels, and their Level Importance Values from the stakeholder questionnaire.

Issues		Levels
Horsemanship	1	All staff are experienced and well trained, employing knowledge of equine behaviour in management and training.
	2	50% of staff are experienced and well trained and sometimes employ knowledge of equine behaviour in management and training.
	3	None of the staff are experienced or well trained, and do not employ knowledge of equine behaviour in management and training.
Health and disease	1	Regular attention to health. Appropriate use of analgesics, tranquilizers, and parasitic control.
	2	Some attention to health. Occasional use of analgesics, tranquilizers and parasitic control medication.
	3	Infrequent attention to health. Analgesics, tranquilizers and parasitic control medication used only when absolutely necessary.
Education of horse	1	Regular training from birth, through weaning, sales preparation and transporting, riding, track work, barrier habituation and racing.
	2	Some handling as a foal, through to weaning, sales preparation and transportation, riding, track work, barrier habituation, and racing.
	3	No handling as a foal or weanling. Little preparation for sales and transporting. Riding and track work rushed with no habituation to the barrier.
Track design and Surface	1	Gradual turning cambered turf track.
	2	Gradual turning cambered synthetic track.
	3	Tight turning cambered turf track.
	4	Tight turning cambered synthetic track.
Ventilation	1	Good ventilation; fans in every stable; good ventilation in transport.
	2	Some ventilation; fans at the end of stable corridors; some ventilation in transport.
	3	Poor ventilation; stable walls of solid construction to 110 cm with wire mesh above; inadequate ventilation in transport.
Stabling	1	Large 5 m × 5 m × 6 m stable with free use of attached yard.
	2	Stable 3.6 m × 3.6 m × 4 m with free use of attached yard.
	3	Stable 5 m × 5 m × 6 m with no use of attached yard.
	4	Stable 3.6 m × 3.6 m × 4 m with no use of attached yard.
Weaning	1	Two weanlings isolated together in a stable which allows visual and physical contact with neighbouring horses.
	2	Removal of one mare at a time from a group of mares and foals in a large paddock, until all mares are removed from the group.
	3	One weanling in a stable which does not allow visual or physical contact with neighbouring horses.
Transport	1	Skilled driver, experienced staff for loading and off-loading horses.
	2	Semi-skilled driver, experienced staff for loading and off-loading horses.
	3	Staff with limited experience in driving, loading and off-loading horses.
Nutrition	1	Attention to age and training requirements of individual horse in order to balance fibre/grain intake, with proven supplement requirements, and access to additional green forage.
	2	Attention to age and training requirements of individual horse in order to balance fibre/grain intake with proven supplement requirements, infrequent access to additional green forage.
	3	Standard nutritional program for all horses regardless of racing program, no additional green forage.
Wastage	1	Horse retired for equestrian sports.
	2	Horse retired from racing to a breeding farm.
	3	Horse given away as race record was insufficient for breeding or temperament unsuitable for retraining in equestrian sports.
	4	Horse sent to a slaughterhouse, unsuitable for further use.
Heat and humidity	1	Horses rarely exposed to climatic variation; some acclimatisation following transport; stable design allows for good temperature control.
	2	Horses sometimes exposed to climatic variation; some acclimatisation following transport; stable design allows for some temperature control.
	3	Horses regularly exposed to climatic variations; inadequate acclimatisation following transport; poor stable design for temperature control.
Whips	1	Whipping the horse occasionally throughout the race.
	2	No use of whip, jockeys ride with hands and heels.
	3	Whipping a tired horse regularly in the last 100 metres of the race.
Environment	1	Use of wood shavings. Stable/yard design allows only visual contact with other horses.
	2	Use of wood shavings. Stable/yard design allows physical and visual contact with other horses.
	3	Use of straw bedding. Stable/yard design allows physical and visual contact with other horses.
	4	Use of straw bedding. Stable/yard design allows only visual contact with other horses.
Gear	1	No blinkers or tongue tie.
	2	Use of blinkers, but no tongue tie.
	3	Use of tongue tie and blinkers.
	4	Use of tongue tie but no blinkers

**Table 2:** Summary of the number of responses from the different stakeholder categories.

Stakeholder	Number Sent	Number Responded	% Stakeholder Response	% of Overall Respondents
Breeders	315	48	15	21
Trainers	793	56	7	25
Owners	26	25	96	11
Veterinarians	135	38	28	17
Transporters	37	4	11	2
Farriers	119	8	7	4
Sales	93	15	16	7
Racing administration	245	21	9	9
TBR Re-trainers	10	9	90	4
TOTAL	1773	224		100

**Table 3:** Respondents’ demographic characteristics (*n* = 224).

Demographic	No of Respondents (%)
Gender	
Male	126 (56.3)
Female	98 (43.8)
Age (years)	
<19	0 (0.0)
19–24	3 (1.3)
25–30	16 (7.1)
31–40	37 (16.5)
41–50	64 (28.6)
51–60	60 (26.8)
>60	44 (19.6)
Highest level of education achieved	
Primary School	5 (2.2)
High School	51 (22.8)
Technical and further education college	56 (25)
University	105 (46.9)
Other	7 (3.1)
Primary involvement with TBR	
Breeder	48 (21)
Farrier	8 (4)
Owner	25 (11)
Practising veterinarian	38 (17)
Re-trainer	9 (4)
Transporter	4 (2)
Trainer	56 (25)
Racing administration	21 (9)
Sales	15 (7)
Duration (months) of practical experience with TBR	
1–12	8 (4)
13–48	12 (5)
over 48	204 (91)

**Table 4:** Median values of rank of each welfare issue, and Wilcoxon sign ranked 95% Confidence Intervals for the 14 issues, as assessed by respondents (*n* = 224).

Welfare Issue	Estimated Median Rank	Confidence Interval	
		Lower	Upper
Horsemanship	8.80	8.60	9.05
Health and disease	8.50	8.25	8.75
Education of horse	8.50	8.25	8.75
Track design and Surface	7.60	7.35	7.90
Ventilation	8.00	7.75	8.25
Weaning	7.55	7.30	7.80
Nutrition	7.45	7.15	7.70
Stabling	7.35	7.05	7.60
Transport	7.25	6.95	7.50
Wastage	7.15	6.80	7.45
Heat and humidity	6.85	6.60	7.10
Whips	6.55	6.20	6.90
Environment	6.00	5.60	6.35
Gear	2.80	2.45	3.05

**Table 5:** Median Importance Value of each issue for respondents with 1–12, 13–48 and >48 months of experience with TBR.

Issues	1–12 Months	13–48 Months	>48 Months	Probability Value
Horsemanship	7.2	8.25	8.9	0.08
Health and Disease	7.1	8.95	8.55	0.31
Education of Horse	6.6	7.75	8.55	0.08
Ventilation	7.25	8.7	8.0	0.06
Track design and Surface	7.1	7.75	7.4	0.66
Stabling	7.15	7.90	7.3	0.66
Weaning	7.15	7.1	7.6	0.41
Transport	6.55	5.7	7.3	0.01
Nutrition	6.05	6.4	7.6	0.04
Wastage	7.15	7.05	7.3	0.10
Heat and Humidity	7.00	7.45	6.8	0.45
Whips	6.85	7.15	6.8	0.80
Environment	8.55	5.05	6.3	0.001
Gear	6.5	5.25	0.9	0.06

## Data Availability

Not applicable.

## Appendix A

**Table A1:** Median Importance Value of each Issue for each stakeholder group.

Issues	Breeder	Farrier	Owner	Practising Vet	Re-Trainer	Transporter	Trainer	Racing Admi	Sales	*p* Value
Horsemanship	9.0	8.5	8.6	8.9	8.7	11.0	8.5	8.9	9.1	0.68
Health and Disease	8.4	7.2	8.2	8.8	8.6	8.0	8.4	8.7	8.9	0.76
Education of horse	8.3	8.6	7.9	8.4	8.8	10.5	8.5	8.2	8.7	0.71
Track Design/Surface	7.4	9.3	7.4	7.2	7.4	6.0	7.9	7.5	7.4	0.64
Ventilation	8.0	8.7	7.1	8.1	8.7	6.9	8.4	7.4	7.6	0.14
Stabling	7.4	7.0	7.5	7.2	9.2	6.8	7.7	7.2	5.6	0.55
Weaning	7.9	7.2	7.5	7.7	6.7	7.9	8.0	7.8	7.0	0.02
Transport	7.3	7.2	7.1	7.4	6.0	9.2	6.7	7.1	6.5	0.28
Nutrition	7.8	5.9	7.9	6.5	6.4	9.1	8.0	7.1	8.1	0.01
Wastage	7.4	7.5	7.0	6.5	6.7	7.0	8.0	7.2	7.4	0.29
Heat and Humidity	6.7	5.8	6.9	7.4	6.6	7.8	6.8	6.8	6.1	0.55
Whips	6.5	7.6	7.1	6.5	7.9	6.3	6.8	6.8	7.7	0.23
Environment	6.5	6.5	6.0	5.7	5.9	5.5	5.8	7.0	6.4	0.85
Gear	0.2	0.6	2.5	1.2	5.6	3.1	1.5	0.3	0.0	0.79

**Table A2:** Level importance values determined from the stakeholder questionnaire.

Issues		Levels	Level Importance Values
Horsemanship	1	All staff are experienced and well trained, employing knowledge of equine behaviour in management and training.	62.29
	2	50% of staff are experienced and well trained and sometimes employ knowledge of equine behaviour in management and training.	−3.89
	3	None of the staff are experienced or well trained, and do not employ knowledge of equine behaviour in management and training.	−58.40
Health and disease	1	Regular attention to health. Appropriate use of analgesics, tranquilizers, and parasitic control.	62.35
	2	Some attention to health. Occasional use of analgesics, tranquilizers and parasitic control medication.	−14.68
	3	Infrequent attention to health. Analgesics, tranquilizers and parasitic control medication used only when absolutely necessary.	−47.66
Education of horse	1	Regular training from birth, through weaning, sales preparation and transporting, riding, track work, barrier habituation and racing.	52.80
	2	Some handling as a foal, through to weaning, sales preparation and transportation, riding, track work, barrier habituation, and racing.	7.38
	3	No handling as a foal or weanling. Little preparation for sales and transporting. Riding and track work rushed with no habituation to the barrier.	−60.18
Track design and Surface	1	Gradual turning cambered turf track.	51.01
	2	Gradual turning cambered synthetic track.	27.03
	3	Tight turning cambered turf track.	−33.12
	4	Tight turning cambered synthetic track.	−44.91
Ventilation	1	Good ventilation; fans in every stable; good ventilation in transport.	48.72
	2	Some ventilation; fans at the end of stable corridors; some ventilation in transport.	6.12
	3	Poor ventilation; stable walls of solid construction to 110 cm with wire mesh above; inadequate ventilation in transport.	−54.84
Stabling	1	Large 5 m × 5 m × 6 m stable with free use of attached yard.	37.72
	2	Stable 3.6 m × 3.6 m × 4 m with free use of attached yard.	14.26
	3	Stable 5 m × 5 m × 6 m with no use of attached yard.	−8.39
	4	Stable 3.6 m × 3.6 m × 4 m with no use of attached yard.	−43.59
Weaning	1	Two weanlings isolated together in a stable which allows visual and physical contact with neighbouring horses.	26.65
	2	Removal of one mare at a time from a group of mares and foals in a large paddock, until all mares are removed from the group.	24.29
	3	One weanling in a stable which does not allow visual or physical contact with neighbouring horses.	−50.94
Transport	1	Skilled driver, experienced staff for loading and off-loading horses.	51.43
	2	Semi-skilled driver, experienced staff for loading and off-loading horses.	−7.98
	3	Staff with limited experience in driving, loading and off-loading horses.	−43.45
Nutrition	1	Attention to age and training requirements of individual horse in order to balance fibre/grain intake, with proven supplement requirements, and access to additional green forage.	47.81
	2	Attention to age and training requirements of individual horse in order to balance fibre/grain intake with proven supplement requirements, infrequent access to additional green forage.	0.85
	3	Standard nutritional program for all horses regardless of racing program, no additional green forage.	−48.66
Wastage	1	Horse retired for equestrian sports.	30.44
	2	Horse retired from racing to a breeding farm.	22.85
	3	Horse given away as race record was insufficient for breeding or temperament unsuitable for retraining in equestrian sports.	−16.37
	4	Horse sent to a slaughterhouse, unsuitable for further use.	−36.92
Heat and humidity	1	Horses rarely exposed to climatic variation; some acclimatisation following transport; stable design allows for good temperature control.	31.25
	2	Horses sometimes exposed to climatic variation; some acclimatisation following transport; stable design allows for some temperature control.	12.37
	3	Horses regularly exposed to climatic variations; inadequate acclimatisation following transport; poor stable design for temperature control.	−43.63
Whips	1	Whipping the horse occasionally throughout the race.	23.71
	2	No use of whip, jockeys ride with hands and heels.	10.91
	3	Whipping a tired horse regularly in the last 100 metres of the race.	−34.61
Environment	1	Use of wood shavings. Stable/yard design allows only visual and physical contact with other horses.	3.44
	2	Use of wood shavings. Stable/yard design allows visual contact with other horses.	0.10
	3	Use of straw bedding. Stable/yard design allows physical and visual contact with other horses.	−1.45
	4	Use of straw bedding. Stable/yard design allows only visual contact with other horses.	−2.09
Gear	1	No blinkers or tongue tie.	14.74
	2	Use of blinkers, but no tongue tie.	1.57
	3	Use of tongue tie and blinkers.	−7.55
	4	Use of tongue tie but no blinkers	−8.76
